# Clinical Effectiveness of Tap Water Versus Povidone-Iodine Bladder Irrigation for Recurrent Catheter-Associated Urinary Tract Infections: A Comparative Review of Contemporary Clinical Evidence

**DOI:** 10.7759/cureus.115123

**Published:** 2026-08-24

**Authors:** Yatin Talwar, Himanshu Agrawal, Kumar Dharmendra Singh, Chandrakant Pilania

**Affiliations:** 1 Hospital Administration, North Eastern Indira Gandhi Regional Institute of Health and Medical Sciences, Shillong, IND; 2 Physical Medicine and Rehabilitation, All India Institute of Medical Sciences, Bilaspur, Bilaspur, IND; 3 Epidemiology and Public Health, All India Institute of Medical Sciences, Raebareli, Raebareli, IND; 4 Physical Medicine and Rehabilitation, All India Institute of Medical Sciences, Bhatinda, Bhatinda, IND

**Keywords:** catheter-associated urinary tract infections, povidone-iodine, recurrent urinary tract infection, urinary bladder irrigation, urinary catheterization

## Abstract

Catheter-associated urinary tract infections (CAUTIs) remain among the most frequent healthcare-associated infections. Increasing antimicrobial resistance has renewed interest in bladder irrigation as a non-antibiotic therapeutic strategy. Tap water and povidone-iodine are two readily available irrigation solutions that have recently been evaluated in prospective clinical studies. A comparative narrative review was undertaken using two prospective clinical investigations published in peer-reviewed journals to compare the clinical effectiveness, safety, tolerability, and potential role of tap-water and povidone-iodine bladder irrigation in patients with recurrent CAUTIs. Study characteristics, patient demographics, intervention protocols, outcome measures, microbiological findings, safety outcomes, quality-of-life measures, and clinical applicability were compared qualitatively. Both bladder irrigation strategies appear clinically beneficial. Povidone-iodine demonstrated greater reductions in infection-related outcomes but involved daily antiseptic instillation in a narrowly defined neurogenic bladder population. Tap-water irrigation produced moderate reductions in antibiotic use and recurrent urinary tract infections while maintaining excellent patient acceptability and quality of life, suggesting an attractive low-cost strategy suitable for wider implementation. Direct comparative randomized trials are required.

## Introduction and background

Catheter-associated urinary tract infection (CAUTI) continues to represent one of the most frequent healthcare-associated infections worldwide despite considerable advances in infection prevention, catheter technology, and antimicrobial therapy. It is estimated that urinary catheterization accounts for approximately three-quarters of all healthcare-associated urinary tract infections (UTIs), making CAUTI a persistent challenge in both acute-care hospitals and long-term healthcare facilities [1,2]. Patients requiring indwelling urethral catheters, suprapubic catheters, or clean intermittent catheterization (CIC) remain particularly vulnerable because urinary catheterization bypasses the natural mechanical and immunological defenses of the lower urinary tract, providing microorganisms direct access to the bladder and facilitating rapid bacterial colonization [2-4]. Consequently, recurrent UTIs remain a major source of morbidity among patients with chronic catheter dependence, particularly those with neurogenic lower urinary tract dysfunction (NLUTD), spinal cord injury, multiple sclerosis, and other neurological disorders requiring lifelong catheterization.

The clinical significance of CAUTI extends well beyond localized UTI. Recurrent infections frequently result in repeated outpatient consultations, emergency department visits, hospital admissions, diagnostic investigations, and multiple courses of antimicrobial therapy, thereby substantially increasing healthcare costs and resource utilization [2,4]. In addition to the direct clinical burden, recurrent catheter-associated infections adversely affect patients' quality of life by causing chronic urinary symptoms, anxiety regarding recurrent illness, interruption of rehabilitation programmes, and reduced independence among patients performing self-catheterization. These cumulative consequences have transformed CAUTI from a seemingly routine healthcare-associated infection into an important public health concern requiring innovative preventive strategies.

The pathogenesis of CAUTI is complex and largely driven by bacterial biofilm formation on catheter surfaces. Following catheter insertion, host proteins rapidly coat both internal and external catheter surfaces, creating an environment conducive to bacterial adhesion. Uropathogens including *Escherichia coli*, *Klebsiella pneumoniae*, *Proteus mirabilis*, *Pseudomonas aeruginosa*, and *Enterococcus* species subsequently attach to these conditioning films and develop structured biofilms embedded within an extracellular polymeric matrix [4,5]. Once established, biofilms provide microorganisms with remarkable protection against host immune responses and systemic antimicrobial therapy. Bacteria residing within mature biofilms may exhibit antimicrobial tolerance many times greater than their planktonic counterparts, allowing persistent colonization despite repeated antibiotic exposure [4]. This biofilm-associated persistence explains why recurrent symptomatic infections frequently recur soon after completion of antimicrobial therapy, particularly among patients requiring long-term catheterization.

An equally important concern is the global emergence of antimicrobial resistance. Repeated empirical antibiotic treatment for recurrent UTIs has accelerated the selection of multidrug-resistant organisms, complicating subsequent treatment and reducing available therapeutic options [6]. Patients with chronic catheterization often receive several antibiotic courses annually, many of which are prescribed for bacteriuria rather than true symptomatic infection. International guidelines increasingly emphasize that asymptomatic bacteriuria in catheterized patients generally should not be treated because antimicrobial therapy offers little clinical benefit while promoting resistance and exposing patients to unnecessary adverse effects [2,3]. Consequently, antimicrobial stewardship programmes now advocate judicious antibiotic prescribing together with development of effective non-antibiotic interventions capable of reducing infection burden without increasing antimicrobial consumption [6,7].

Within this context, bladder irrigation has re-emerged as a promising adjunctive strategy. Historically, bladder irrigation was primarily employed to maintain catheter patency, evacuate blood clots following urological surgery, and prevent catheter obstruction. Over subsequent decades, investigators explored the use of various irrigation solutions, including saline, antiseptics, antibiotics, acidic preparations, and more recently tap water, to reduce bacterial colonization and prevent recurrent infection [8,9]. Although earlier studies yielded inconsistent results and enthusiasm gradually declined following concerns regarding routine antimicrobial irrigation, the contemporary emphasis on antimicrobial stewardship has renewed interest in simple, non-antibiotic interventions capable of reducing recurrent symptomatic infection.

The theoretical rationale for bladder irrigation differs according to the solution employed. Mechanical irrigation using tap water aims to reduce bacterial burden through repeated flushing of infected urine, mucus, inflammatory debris, and early biofilm components without exposing microorganisms to antimicrobial agents. By contrast, antiseptic irrigation with povidone-iodine combines mechanical lavage with broad-spectrum antimicrobial activity against bacteria, fungi, viruses, and protozoa while exhibiting minimal potential for inducing bacterial resistance [9,10]. These fundamentally different mechanisms provide an opportunity to compare whether mechanical clearance alone is sufficient to improve clinical outcomes or whether additional antiseptic activity confers measurable clinical benefit.

Interest in bladder irrigation has intensified following two prospective clinical investigations published in recent years. Moussa and colleagues evaluated daily intravesical povidone-iodine irrigation in patients with neurogenic bladder managed by clean intermittent catheterization and demonstrated remarkable reductions in recurrent symptomatic UTIs, emergency department attendance, hospitalizations, and multidrug-resistant urinary isolates [10]. More recently, den Hoedt and colleagues investigated patient-administered tap-water bladder irrigation among community catheter users and reported significant reductions in antibiotic use and recurrent UTIs while maintaining excellent patient satisfaction and stable quality of life [11,12]. These studies represent important advances because they move beyond historical anecdotal experience and provide prospective clinical evidence supporting bladder irrigation as a potentially valuable component of contemporary CAUTI management.

Despite increasing interest, important uncertainties remain regarding the comparative role of these interventions in routine clinical practice. The available studies differ considerably in patient selection, catheter type, irrigation technique, treatment frequency, outcome measures, and duration of follow-up, making direct comparison challenging. Furthermore, current international guidelines continue to provide limited recommendations regarding bladder irrigation because robust randomized comparative evidence remains scarce [13-15]. Consequently, clinicians are faced with growing interest in bladder irrigation but relatively limited guidance regarding patient selection, choice of irrigation solution, expected clinical benefit, and implementation within antimicrobial stewardship programmes.

The present narrative review, based on two prospective studies, therefore critically examines the contemporary evidence supporting tap-water and povidone-iodine bladder irrigation for recurrent catheter-associated UTIs. Rather than simply comparing individual study findings, this review synthesizes current knowledge regarding the biological rationale, clinical effectiveness, safety, microbiological outcomes, patient-centred outcomes, and practical implementation of both interventions. Particular emphasis is placed on identifying the respective strengths and limitations of the available evidence, evaluating the contribution of bladder irrigation to antimicrobial stewardship, and highlighting areas requiring future investigation. By integrating recent prospective clinical studies with current international guidance and established knowledge regarding catheter-associated UTIs, this review aims to provide clinicians with a balanced and clinically relevant appraisal of the evolving role of bladder irrigation in modern urological practice.

## Review

Biological rationale for bladder irrigation

The renewed interest in bladder irrigation reflects a broader paradigm shift in the management of recurrent CAUTIs. Historically, treatment strategies have relied predominantly on repeated courses of systemic antibiotics, with bladder irrigation largely confined to maintaining catheter patency or evacuating blood clots following urological procedures. However, increasing recognition of antimicrobial resistance, together with an improved understanding of biofilm biology, has prompted reconsideration of local intravesical therapies as adjunctive strategies capable of reducing bacterial burden while limiting systemic antibiotic exposure [2,4,6].

The development of CAUTI is intrinsically linked to bacterial biofilm formation on catheter surfaces. Following catheter insertion, host proteins rapidly coat both the internal and external catheter surfaces, creating an ideal substrate for bacterial adhesion. Microorganisms subsequently proliferate within an extracellular polymeric matrix composed of polysaccharides, proteins, nucleic acids, and cellular debris, forming highly structured biofilms that are remarkably resistant to host immune responses and antimicrobial penetration [4,5]. Mature biofilms function as persistent microbial reservoirs, allowing bacteria to survive prolonged antibiotic exposure and repeatedly seed planktonic organisms into the bladder lumen, thereby perpetuating recurrent symptomatic infection. This biological phenomenon largely explains why eradication of bacteriuria is frequently unsuccessful in patients requiring chronic catheterization and why recurrent infection often occurs shortly after completion of apparently appropriate antimicrobial therapy.

Bladder irrigation attempts to interrupt this pathogenic cycle through local intravesical intervention. Unlike systemic antibiotics, which depend upon adequate tissue penetration and bacterial susceptibility, irrigation directly targets the bladder lumen and catheter surfaces. The anticipated benefits include mechanical removal of contaminated urine, dilution of inflammatory debris, reduction in bacterial inoculum, disruption of immature biofilms, and prevention of prolonged urinary stasis. These effects are particularly relevant among patients with incomplete bladder emptying, neurogenic bladder dysfunction, or chronic catheterization, where urinary stagnation promotes persistent bacterial colonization [2,4].

Although both tap water and povidone-iodine employ bladder irrigation as the therapeutic principle, their mechanisms differ fundamentally. Tap-water irrigation functions primarily through mechanical lavage. Repeated instillation and aspiration physically remove bacteria, mucus, exfoliated epithelial cells, inflammatory exudate, and loosely adherent biofilm components. Importantly, because the intervention contains no antimicrobial agent, it exerts minimal selective pressure for bacterial resistance. This characteristic aligns closely with contemporary antimicrobial stewardship principles, which encourage non-antibiotic approaches whenever clinically appropriate [6,7]. Moreover, tap water is inexpensive, universally available, and can be administered independently by patients after appropriate training, making it particularly attractive for long-term outpatient management.

Povidone-iodine irrigation, in contrast, combines mechanical irrigation with potent local antiseptic activity. Povidone-iodine possesses broad-spectrum antimicrobial efficacy against Gram-positive and Gram-negative bacteria, fungi, viruses, and protozoa through oxidation and iodination of microbial proteins, resulting in rapid disruption of cellular metabolism and membrane integrity. Unlike antibiotics, povidone-iodine acts through multiple nonspecific biochemical mechanisms, thereby substantially reducing the likelihood of acquired microbial resistance. This broad antimicrobial spectrum provides a plausible explanation for the marked reduction in positive urine cultures and multidrug-resistant isolates observed in the prospective neurogenic bladder study [10]. Nevertheless, prolonged antiseptic exposure also raises theoretical concerns regarding local urothelial irritation, iodine absorption, and thyroid dysfunction, necessitating careful evaluation of safety in long-term use.

These contrasting mechanisms suggest that tap water and povidone-iodine should not necessarily be viewed as competing interventions but rather as therapies potentially suited to different clinical scenarios. Mechanical irrigation may adequately reduce bacterial burden among patients with uncomplicated recurrent infections, whereas antiseptic irrigation may provide additional benefit in carefully selected high-risk populations characterized by persistent biofilm formation and multidrug-resistant colonization.

Contemporary clinical evidence

The contemporary evidence supporting bladder irrigation remains limited but has improved considerably following the publication of two prospective clinical investigations that evaluated fundamentally different irrigation strategies. Although these studies are frequently discussed together, careful interpretation requires recognition that they addressed different patient populations, therapeutic objectives, and outcome measures. Consequently, direct numerical comparison should be approached cautiously.

Moussa and colleagues evaluated daily intravesical povidone-iodine irrigation among 119 adults with NLUTD managed by clean intermittent catheterization who experienced recurrent symptomatic UTIs despite optimized bladder management [10]. The study population represented a relatively young, highly selected cohort with severe underlying neurological disease, predominantly spinal cord injury. Patients received daily instillation of 50 mL of 2% povidone-iodine as the final catheterization of the day, with the solution retained intravesically for approximately 10 minutes before drainage. Monthly biochemical surveillance was performed to monitor serum iodine concentrations and thyroid function, reflecting appropriate consideration of potential systemic absorption.

The investigators reported substantial reductions in symptomatic UTIs, emergency department attendance, hospitalization, and multidrug-resistant urinary isolates during follow-up. These improvements remained statistically significant following multivariable adjustment, suggesting that the observed treatment effect could not be fully explained by demographic or disease-related confounding [10]. From a clinical perspective, the magnitude of benefit was striking and suggested that local antiseptic therapy may effectively interrupt recurrent infection cycles among carefully selected neurogenic bladder patients.

In contrast, den Hoedt and colleagues investigated a substantially broader patient population more representative of routine outpatient urological practice [11]. Their prospective study enrolled 60 community-dwelling catheter users, including both patients performing clean intermittent catheterization and individuals managed with long-term transurethral or suprapubic indwelling catheters. Unlike the neurogenic bladder cohort studied by Moussa et al., nearly half of these participants had non-neurogenic indications for catheterization, resulting in greater clinical heterogeneity and potentially lower baseline infection risk.

The intervention itself also reflected a different therapeutic philosophy. Rather than scheduled daily prophylaxis, patients initiated tap-water irrigation at the earliest onset of uncomplicated urinary tract symptoms using warmed tap water and repeated irrigation until the returned fluid appeared macroscopically clear. Irrigation frequency was subsequently reduced according to symptomatic improvement. This patient-directed approach emphasized self-management, symptom control, and avoidance of unnecessary antibiotic treatment rather than complete microbiological eradication.

The principal outcome was reduction in antibiotic consumption rather than prevention of bacteriuria. Investigators demonstrated approximately 38% reductions in both antibiotic prescriptions and recurrent UTIs without increased hospitalization, systemic infection, or deterioration in health-related quality of life [11]. Importantly, patient satisfaction exceeded 80% across multiple domains, indicating that the procedure was considered practical, acceptable, and feasible for long-term home use.

These two investigations therefore evaluated different clinical questions rather than identical interventions. The povidone-iodine study primarily addressed whether scheduled antiseptic bladder irrigation could prevent recurrent infection among a highly selected high-risk neurogenic population. Conversely, the tap-water study examined whether mechanical bladder irrigation could reduce antibiotic consumption among a heterogeneous outpatient catheter population. Consequently, interpretation should emphasize complementary rather than competitive evidence.

Several methodological strengths deserve recognition. Both studies were prospective, incorporated clinically meaningful outcomes, and evaluated interventions readily translatable into routine practice. Importantly, neither investigation reported serious safety concerns attributable to bladder irrigation, supporting the feasibility of carefully supervised intravesical therapy.

Nevertheless, several limitations substantially influence interpretation. First, both studies enrolled relatively small numbers of participants, limiting statistical precision and external validity. Second, neither investigation was designed as a direct randomized comparison between irrigation solutions. Third, considerable differences in patient demographics, catheter characteristics, intervention protocols, and primary endpoints preclude meaningful quantitative comparison. Finally, follow-up durations were relatively limited, leaving uncertainty regarding long-term effectiveness, durability of response, and sustainability of patient adherence.

Collectively, the available evidence supports the concept that bladder irrigation may reduce recurrent symptomatic UTIs while decreasing reliance on systemic antimicrobial therapy. However, current data are insufficient to conclude that one irrigation solution is unequivocally superior. Instead, both interventions appear to occupy distinct yet complementary positions within contemporary management strategies for recurrent CAUTIs. 

Clinical effectiveness

The principal objective of bladder irrigation is to reduce the frequency and severity of recurrent CAUTIs while minimizing dependence on systemic antimicrobial therapy. Although both contemporary prospective studies demonstrated favourable clinical outcomes, they evaluated different patient populations, intervention strategies, and therapeutic objectives, making careful interpretation essential.

The most striking clinical improvements were reported by Moussa et al., who observed dramatic reductions in symptomatic UTIs, emergency department attendance, and UTI-related hospitalizations following daily intravesical povidone-iodine irrigation in patients with NLUTD performing clean intermittent catheterization [10]. The magnitude of reduction suggests that regular antiseptic irrigation may effectively suppress recurrent bacterial colonization in carefully selected high-risk patients who continue to experience infections despite optimized catheterization practices. The concurrent decline in healthcare utilization further indicates that the intervention may reduce not only infection frequency but also infection severity.

The tap-water study by den Hoedt et al. demonstrated more modest but clinically meaningful improvements [11]. Approximately one-third fewer antibiotic prescriptions and recurrent UTIs were observed without an increase in pyelonephritis, systemic infection, or hospitalization. Importantly, these findings suggest that a proportion of uncomplicated recurrent urinary symptoms may be successfully managed through local bladder irrigation rather than immediate empirical antibiotic therapy. This observation is particularly relevant in the current era of antimicrobial stewardship, where avoiding unnecessary antibiotic exposure is considered a major therapeutic objective.

Direct comparison of treatment efficacy between the two interventions is neither statistically nor clinically appropriate. The neurogenic bladder population investigated by Moussa et al. represented a younger, highly selected cohort with severe recurrent infections and uniform catheterization technique, whereas the study by den Hoedt et al. included older patients with heterogeneous indications for catheterization and mixed catheter types [10,11]. Baseline infection risk, underlying urinary tract pathology, and treatment objectives therefore differed substantially between the investigations. Consequently, the greater reductions observed with povidone-iodine should not automatically be interpreted as evidence of superiority but rather as reflecting differences in patient selection and clinical context.

Collectively, the available evidence suggests that bladder irrigation may provide clinically relevant reductions in recurrent infection burden when incorporated into comprehensive catheter management strategies. However, the current literature supports individualized patient selection rather than universal adoption of either irrigation strategy.

Safety and tolerability

Long-term safety remains a fundamental consideration for any intervention intended for chronic catheter users. Because bladder irrigation often requires repeated administration over months or years, acceptable tolerability is essential to ensure sustained adherence and clinical benefit.

Both prospective studies reported favourable safety profiles, although the nature of safety monitoring differed considerably. The povidone-iodine investigation incorporated regular biochemical surveillance, including serum iodine concentrations and thyroid-stimulating hormone measurements, reflecting concern regarding potential systemic iodine absorption following repeated intravesical administration [10]. Reassuringly, no clinically significant thyroid dysfunction or evidence of iodine toxicity was observed during follow-up. Adverse events were generally mild and consisted predominantly of transient lower urinary tract irritation, occasional genital discomfort, and infrequent episodes of mild hematuria. No participant experienced severe complications directly attributable to the irrigation protocol.

Similarly, tap-water irrigation demonstrated excellent overall tolerability [11]. No increase in upper UTI, sepsis, or hospitalization was observed despite substantial reductions in antibiotic use. Most participants successfully performed bladder irrigation independently at home after receiving standardized instruction, and only a small proportion discontinued treatment because of procedural inconvenience or discomfort. Stable health-related quality-of-life scores further support the conclusion that the intervention did not adversely affect patients' overall well-being.

Although these findings are encouraging, several limitations deserve consideration. Neither study evaluated very long-term outcomes extending beyond routine follow-up, and both involved relatively small sample sizes. Consequently, uncommon adverse events may have remained undetected. Furthermore, the safety of repeated tap-water irrigation may vary according to local water quality, emphasizing the importance of patient education regarding appropriate technique and use of clean water sources. Likewise, although no thyroid abnormalities were identified following povidone-iodine irrigation, continued caution may be warranted in patients with pre-existing thyroid disease, pregnancy, or prolonged exposure.

Overall, current evidence supports the short-term safety of both irrigation strategies when undertaken using standardized techniques and appropriate clinical supervision. Nevertheless, larger studies with extended follow-up remain necessary to establish long-term safety with greater certainty.

Microbiological outcomes and antimicrobial stewardship

One of the most important contemporary justifications for bladder irrigation is its potential contribution to antimicrobial stewardship. The widespread emergence of multidrug-resistant uropathogens has highlighted the need for effective non-antibiotic interventions capable of reducing infection burden while preserving antibiotic efficacy [3,6,7].

The microbiological evidence currently favours povidone-iodine irrigation. Moussa et al. demonstrated significant reductions in positive urine cultures together with a marked decline in multidrug-resistant urinary isolates following treatment [10]. These findings are biologically plausible given the broad-spectrum antiseptic activity of povidone-iodine and its multiple nonspecific mechanisms of microbial inactivation. Unlike conventional antibiotics, antiseptics exert limited selective pressure for resistance, making them attractive adjuncts in the management of recurrent infections caused by resistant organisms.

By contrast, the tap-water study deliberately emphasized clinical rather than microbiological outcomes [11]. Routine urine cultures were not used as primary outcome measures because the investigators adopted a symptom-based management strategy consistent with contemporary guideline recommendations that discourage treatment of asymptomatic bacteriuria in catheterized patients [2,3]. This pragmatic approach reflects an important conceptual shift in CAUTI management. Complete microbiological eradication is often unrealistic in chronically catheterized patients because biofilm-associated bacteriuria frequently persists despite apparently appropriate antimicrobial therapy. Instead, improving patient symptoms while reducing unnecessary antibiotic exposure may represent a more clinically meaningful therapeutic objective.

The reduction in antibiotic prescriptions observed following tap-water irrigation therefore represents a particularly important finding. Fewer antibiotic courses may translate into reduced selection pressure for antimicrobial resistance, lower incidence of antibiotic-associated adverse events, and decreased healthcare costs. Although direct evidence linking tap-water irrigation with reduced multidrug-resistant organisms is currently unavailable, the observed reduction in antimicrobial exposure strongly supports its potential role within broader antimicrobial stewardship programmes.

Taken together, the available evidence suggests that both irrigation strategies may contribute to stewardship through different mechanisms. Povidone-iodine primarily reduces bacterial burden through local antiseptic activity, whereas tap water decreases antibiotic exposure by facilitating symptom-directed conservative management. These complementary mechanisms reinforce the concept that bladder irrigation should be viewed as an adjunct to, rather than a replacement for, established evidence-based infection prevention strategies.

Patient-reported outcomes and practical implementation

Successful long-term management of recurrent CAUTIs depends not only upon microbiological efficacy but also upon patient acceptance, ease of administration, and sustainability of treatment. Interventions perceived as burdensome or uncomfortable are unlikely to achieve satisfactory long-term adherence, particularly among patients requiring lifelong catheterization.

Among the available studies, den Hoedt et al. provided the most comprehensive evaluation of patient-centred outcomes [11]. Treatment satisfaction exceeded 80% across effectiveness, convenience, and overall satisfaction domains, while health-related quality-of-life scores remained stable throughout follow-up. These findings indicate that self-administered tap-water irrigation was generally well accepted and did not impose significant treatment burden. The simplicity of the procedure, minimal equipment requirements, and negligible financial cost may all contribute to favourable long-term adherence, particularly in community-based settings [16-20].

Although standardized patient-reported outcome measures were not formally incorporated into the povidone-iodine study, treatment completion rates and relatively low incidence of clinically significant adverse events suggest satisfactory acceptability among participants [10]. Nevertheless, daily antiseptic instillation, mandatory aseptic technique, and periodic laboratory monitoring may limit its practicality outside specialized neuro-urological services.

Implementation in routine clinical practice should therefore consider individual patient characteristics, healthcare resources, and treatment objectives. Tap-water irrigation appears particularly suitable for motivated patients capable of performing self-care and seeking to reduce recurrent antibiotic use. Povidone-iodine irrigation may be more appropriate for carefully selected high-risk patients managed within specialist centres where structured education and follow-up are available.

Critical appraisal and future directions

In summary, povidone-iodine irrigation combines mechanical lavage with broad-spectrum antiseptic activity and has demonstrated substantial reductions in recurrent symptomatic UTIs, infection-related healthcare utilization, and multidrug-resistant urinary isolates among carefully selected patients with NLUTD performing clean intermittent catheterization. These findings suggest that antiseptic bladder irrigation may represent an effective option for patients experiencing persistent recurrent infections despite optimized bladder management, particularly within specialized neuro-urological practice. Conversely, tap-water bladder irrigation represents a fundamentally different treatment philosophy centred on mechanical removal of contaminated urine, inflammatory debris, and early biofilm components without exposing patients to additional antimicrobial agents. Although the observed reductions in recurrent infection were more modest, the intervention demonstrated significant decreases in antibiotic utilization while maintaining excellent patient satisfaction, favourable tolerability, and stable health-related quality of life. Its simplicity, negligible cost, ease of self-administration, and minimal infrastructure requirements make tap-water irrigation particularly attractive for long-term community management and for integration into antimicrobial stewardship programmes.

Although recent prospective investigations have substantially strengthened the evidence supporting bladder irrigation, important methodological limitations continue to restrict definitive clinical recommendations. The evidence base currently consists primarily of relatively small prospective studies with heterogeneous patient populations, differing catheter types, variable irrigation protocols, and distinct primary outcomes [10,11]. Consequently, direct quantitative comparison between interventions remains inappropriate.

The absence of randomized head-to-head comparisons represents the most important evidence gap. Future multicentre randomized controlled trials should directly compare tap water, povidone-iodine, saline, and other irrigation strategies using standardized definitions of symptomatic UTI, harmonized outcome measures, and sufficiently prolonged follow-up. Such studies should incorporate microbiological surveillance, patient-reported outcomes, health-economic evaluation, and antimicrobial consumption to provide a comprehensive assessment of therapeutic benefit.

Additional research should also identify patient subgroups most likely to benefit from individual irrigation strategies. Patients with neurogenic bladder dysfunction, recurrent multidrug-resistant infections, chronic indwelling catheters, and frequent antibiotic exposure may not derive equivalent benefit from identical interventions. Precision-based patient selection may therefore become increasingly important as evidence evolves.

Finally, bladder irrigation should continue to be evaluated as one component of comprehensive catheter management rather than an isolated intervention. Appropriate catheter indication, minimization of catheter duration, meticulous catheter care, optimization of bladder emptying, and evidence-based antimicrobial prescribing remain the foundation of CAUTI prevention. Bladder irrigation should complement, not replace, these established preventive measures.

## Conclusions

CAUTIs remain among the most challenging complications of long-term urinary catheterization because of their close association with biofilm formation, recurrent symptomatic infection, repeated antimicrobial exposure, and the emergence of multidrug-resistant organisms. Contemporary management has progressively shifted from treating every episode of bacteriuria toward preventing clinically significant infection while minimizing unnecessary antibiotic use. Within this evolving paradigm, bladder irrigation has re-emerged as a promising adjunctive intervention that addresses the underlying intravesical environment rather than relying exclusively on repeated systemic antimicrobial therapy. The current evidence indicates that both tap-water and povidone-iodine bladder irrigation provide clinically meaningful benefits in appropriately selected catheterized patients, although their therapeutic roles appear to differ. Importantly, the available evidence should not be interpreted as demonstrating superiority of one irrigation strategy over the other. Rather than competing approaches, tap-water and povidone-iodine irrigation should be regarded as complementary strategies whose application should be individualized according to patient characteristics, underlying bladder dysfunction, infection severity, catheter type, and available clinical resources.

Despite encouraging findings, the current evidence base remains limited. Most available data originate from relatively small prospective investigations with heterogeneous study populations and limited follow-up. High-quality randomized controlled trials directly comparing irrigation solutions using standardized protocols, uniform outcome definitions, microbiological surveillance, patient-reported outcome measures, and health-economic analyses are still lacking. Such studies are essential before bladder irrigation can be incorporated into routine international clinical practice guidelines. Bladder irrigation represents a promising non-antibiotic adjunct in the management of recurrent CAUTIs, with both tap-water and povidone-iodine irrigation reducing recurrent infection burden while potentially decreasing dependence on systemic antibiotics. Their greatest value may lie not in replacing established preventive measures but in complementing comprehensive catheter management strategies that emphasize appropriate catheter use, meticulous catheter care, optimized bladder drainage, and responsible antimicrobial prescribing. As antimicrobial resistance continues to challenge conventional treatment paradigms, carefully selected bladder irrigation strategies may become increasingly important components of individualized, patient-centred, and stewardship-oriented urological care.
